# Equine Infectious Anemia Virus in Equids: A Large-Scale Serosurvey in Western Europe

**DOI:** 10.3390/ani15233499

**Published:** 2025-12-04

**Authors:** Moisés Gonzálvez, Juan J. Franco, David Cano-Terriza, Jesús Barbero-Moyano, Eduard Jose-Cunilleras, Jesús García, Eduardo Alguacil, Ignacio García-Bocanegra

**Affiliations:** 1 Departamento de Sanidad Animal, Grupo de Investigación en Sanidad Animal y Zoonosis (GISAZ), UIC Zoonosis y Enfermedades Emergentes ENZOEM, Universidad de Córdoba, 14014 Córdoba, Spain; moises.gonzalvez@uclm.es (M.G.); jbarbero962019@gmail.com (J.B.-M.); nacho.garcia@uco.es (I.G.-B.); 2 Grupo Sanidad y Biotecnología (SaBio), Instituto de Investigación en Recursos Cinegéticos (IREC), CSIC-UCLM-JCCM, 13005 Ciudad Real, Spain; 3 Gold Standard Diagnostics Madrid, 28037 Madrid, Spain; jjfpueyo@gmail.com; 4 CIBERINFEC, ISCIII CIBER de Enfermedades Infecciosas, Instituto de Salud Carlos III, 28029 Madrid, Spain; 5 Servei de Medicina Interna Equina, Departament de Medicina i Cirurgia Animals, Facultat de Veterinària, Universitat Autònoma de Barcelona, 08193 Barcelona, Spain; eduard.jose.cunilleras@uab.cat; 6 Fethard Equine Hospital, E91 Y6T8 Tipperary, Ireland; jgmjesus90@gmail.com; 7 Uplands Way Vets, Diss IP22 2AA, UK; eduardoalguacil@hotmail.com

**Keywords:** donkey, Equine Infectious Anemia, Europe, horse, Lentivirus, mule

## Abstract

Background: Equine Infectious Anemia (EIA) is a notifiable disease caused by Equine Infectious Anemia Virus (EIAV), with major sanitary and economic importance for equids worldwide. There is limited and outdated information on the circulation of EIAV in many European countries. In the present study, we aimed to assess virus exposure in different equid species in Western Europe. Methods: Between 2011 and 2023, a cross-sectional study was conducted to investigate the seroprevalence of EIAV in 1676 equids (1444 horses, 106 donkeys and 126 mules/hinnies) from four European regions: Andalusia (Southern Spain; *n* = 808), Catalonia (Northeastern Spain; *n* = 437), Southeastern United Kingdom (UK; *n* = 209), and Ireland (*n* = 222). Results: None of the analyzed equines tested positive for antibodies against EIAV (0%; 95 %CI: 0.0–0.18), indicating limited virus exposure of the equid populations from these European regions. Conclusions: This finding is consistent with the low EIAV circulation previously reported in Western Europe and the few official EIA outbreaks recorded in the region during the last decade. To the best of the author’s knowledge, this serosurvey represents the first investigation assessing EIAV exposure in equids from Ireland and the UK. Our results not only provide novel epidemiological evidence for the four European regions assessed but also reinforce the effectiveness of surveillance strategies and control measures implemented by European countries, in accordance with national and international regulations, to successfully limit EIAV circulation.

## Simple Summary

Equine Infectious Anemia (EIA) is a notifiable disease caused by Equine Infectious Anemia Virus (EIAV), posing significant sanitary and economic risks to equids worldwide. Data on EIAV circulation in Western Europe remain scarce and outdated. This cross-sectional study assessed EIAV seroprevalence in 1676 equids (1444 horses, 106 donkeys, and 126 mules/hinnies) from Southern Spain, Northeastern Spain, Southeastern United Kingdom, and Ireland between 2011 and 2023. We did not detect antibodies against EIAV in the equids analyzed. Our results are in line with those previously reported in Western Europe and support the effectiveness of current surveillance and control measures implemented in accordance with national and international regulations to successfully limit EIAV circulation.

## 1. Introduction

Equine Infectious Anemia (EIA) is a notifiable infectious disease (category D + E; Regulation (EU) 2018/1882) caused by an enveloped single-stranded RNA virus of the family Retroviridae (*Lentivirus equinfane*), hereinafter Equine Infectious Anemia Virus (EIAV), affecting Equidae species. When evaluating the potential transmission routes of EIAV, it is important to consider the type of environment in which equids are maintained. In more natural or extensive systems, where animals inhabit less human-modified environments, transmission mainly occurs through the bites of blood-feeding insects such as horseflies, deerflies, and stable flies [1]. In contrast, in intensive or human-managed systems, iatrogenic transmission could be an important route of infection, particularly when management practices are not properly controlled [1,2]. Although vertical EIAV infection has also been suggested, it appears to be an infrequent transmission pathway [3].

Equine Infectious Anemia Virus exhibits a pronounced tropism for myeloid cells of the monocyte-macrophage lineage, where high expression of the receptor “Equine Lentivirus Receptor 1” facilitates viral entry and interactions with host cells [4]. Although horses (*Equus ferus caballus*), donkeys (*Equus africanus asinus*), and mules (*E. africanus × ferus*) are susceptible to EIAV infection, it has been suggested that donkeys often remain largely asymptomatic compared to horses [5]. Clinical signs span a broad spectrum, ranging from acute or subclinical infection to, in severe cases, death. Affected animals can exhibit various hematological alterations and clinical findings including thrombocytopenia, anemia, recurrent febrile episodes, weight loss, and, occasionally, neurological manifestations [1,6]. However, these cyclical signs generally decrease in both intensity and frequency during the first year post-infection. At this stage, many animals become lifelong carriers with persistent infection [2]. These asymptomatic equids can maintain low levels of circulating EIAV in their blood without showing clinical signs, thereby acting as reservoirs of the virus and enabling transmission by competent vectors [7].

Persistently infected equids play a pivotal epidemiological role in the maintenance and transmission of EIAV within equine populations over time [1,8]. The lack of effective treatments for EIA significantly contributes to the disease’s high economic impact, which primarily stems from the euthanasia of EIAV-positive animals, movement restrictions, and trade limitations [6,9]. Only supportive therapy is available for EIAV-infected animals, aimed at managing febrile episodes and associated clinical signs [6]. Historically, several vaccination programs have been implemented; notably, an attenuated EIAV vaccine used in China during the 1970s demonstrated substantial efficacy in reducing EIAV prevalence [10]. However, current EIA control strategies have shifted from vaccination to animal quarantine to avoid interferences of vaccination with diagnostics testing [11].

After infection, equids may take between 14 and 28 days to seroconvert, and even 38 to 87 days to develop neutralizing antibodies, which poses a relevant challenge for the early detection and management of EIAV-infected individuals [1]. Consequently, although serological tests, such as Agar Gel Immunodiffusion (AGID) or commercial ELISAs targeting proteins p26, gp45, and/or gp90, are widely accepted for screening equine populations [12,13], a broad range of molecular diagnostic protocols has also been implemented to improve detection sensitivity [14,15]. However, ELISA tests represent highly effective diagnostic tools for detecting EIAV circulation and are therefore considered valuable for the control and eradication of EIA [16].

Since the first case of EIA was described in France in 1843 [17], the virus has spread globally, with outbreaks reported in multiple countries across different continents. In Europe, EIAV distribution is generally limited. Countries such as Italy and Romania have historically been considered endemic for EIAV. These countries have implemented robust surveillance programs focused on detecting EIAV-positive animals, followed by their culling or isolation in sanctuaries, which has contributed to reducing EIAV prevalence in these regions [9,18]. In contrast, epidemiological studies in non-endemic European countries have revealed heterogeneous spatial patterns of EIAV circulation. While exposure of equids to EIAV has not been detected in certain European regions, including Switzerland, Bulgaria, Spain, and Poland [19,20,21,22], other countries, particularly in Southeastern Europe, have reported low to moderate EIAV exposure (3.2–12.3%) [23,24,25]. Regarding EIA outbreaks, data recorded by the World Animal Health Information System (WAHIS) of the World Organisation for Animal Health (WOAH) have recorded widespread but spatially heterogeneous notifications across Europe over the last decades [26]. While some countries, such as France (*n* = 37) and Germany (*n* = 82), have reported a relatively high number of EIA outbreaks, others, including Spain, the UK, and Switzerland, have documented only sporadic cases (*n* ≤ 6) [26]. These differences among regions have also been documented by the International Collating Centre (ICC), which has been monitoring equine infectious diseases from January 2019. In this context, the ICC has reported outbreaks of EIA in some countries, such as Italy (*n* = 43), France (*n* = 11), and Bulgaria (*n* = 6), whereas others, including Spain and the UK, have not reported any cases during the same period [27].

Nowadays, up-to-date information on equid exposure to EIAV is lacking in many European countries and regions, such as the UK, Ireland, Portugal, and other central and eastern areas [2]. Consequently, this study aimed to evaluate EIAV exposure on a large scale among equid populations across four regions from Western Europe: Andalusia and Catalonia from Spain, the UK, and Ireland.

## 2. Materials and Methods

### 2.1. Study Area and Sampling

**Figure 1:**
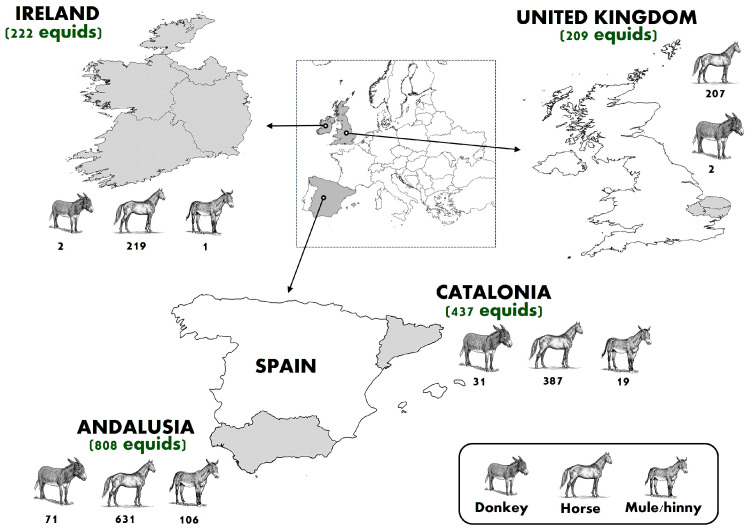
Sample size of each equid species (horses, donkeys, and mules/hinnies) and study region included in the serosurvey. The map shows the geographic distribution of sampled equids across selected regions of Western Europe, namely Ireland, the United Kingdom, and two Spanish regions (Andalusia and Catalonia). The total number of equids sampled by region is shown in parentheses, and the sample size by equid species and region is indicated below each corresponding figure. Figure adapted from [29].

Between 2011 and 2023, a cross-sectional study was carried out to evaluate the prevalence of antibodies against EIAV in 1676 equids from Andalusia (*n* = 808) and Catalonia (*n* = 437) (Southern and Northeastern Spain, respectively), Southeastern UK (n = 209), and Ireland (*n* = 222) (Figure 1). Three different equid species, including horses (*n* = 1444), donkeys (*n* = 106), and mules/hinnies (*n* = 126), were sampled (Table 1). A convenience sampling strategy was employed to obtain serum samples. These samples were collected either during ongoing epidemiological surveillance programs or were submitted as part of routine veterinary check-ups conducted during the study period. Specifically, equine serum samples from the Spanish regions of Andalusia and Catalonia were obtained through the West Nile virus regional surveillance programs, which have been implemented in Spain for several years under the coordination of the Ministry of Agriculture, Fisheries and Food [28]. Samples from Ireland and the UK were sourced from serum banks of private veterinary hospitals, consisting of leftover samples collected after routine biochemical analyses conducted for clinical purposes. All samples were collected in accordance with ethical guidelines, with owners providing prior informed consent for the use of anonymized biological material, including blood, for research purposes. Whenever possible, individual information was also obtained through interviews with equid owners, including age (foal, adult, geriatric), sex (male, female), breed (pure, crossbreed), activity (sport, leisure, reproduction, work), and body condition (overweight, adequate, thin).

For all equids included in the study, blood samples were collected by jugular venipuncture using plain tubes without anticoagulant (Vacutainer^®^, Becton-Dickinson, Franklin Lakes, NJ, USA), which were then centrifuged at 400 g for 10 min and stored at −20 °C until laboratorial analyses.

### 2.2. Serological and Statistical Analysis

Based on previous research studies evaluating the diagnostic performance of EIAV detection methods [16,30], equine serum samples were analyzed for antibodies against EIAV using a commercial double-antigen enzyme-linked immunosorbent assay (ELISA) (Ingezim Anemia Equina, R.14.AIE.K.0, Gold Standard Diagnostics Madrid, Madrid, Spain), which detects specific EIAV antibodies targeting the p26 protein. The ELISA was performed according to the manufacturer’s instructions.

Briefly, microplates pre-coated with recombinant EIAV p26 antigen were incubated with the equid sera samples. After incubation, the plates were washed to remove non-specifically bound components. A horseradish peroxidase (HRPO)-conjugated recombinant p26 protein was then added to allow binding of equine immunoglobulins present in EIAV-positive samples. After a second incubation, the plates were washed again to remove unbound conjugate. Finally, after adding the specific substrate, a colorimetric reaction developed in positive samples, indicating the presence of EIAV antibodies. Absorbance of each sample was measured at 450 nm to determine optical density values. Samples were considered positive when the “sample optical density” to “positive control optical density” was ≥0.3. All remaining samples were classified as negative.

The positive controls included in the ELISA kits were derived from EIAV-positive equids provided by the Instituto Nacional de Tecnología Agropecuaria (Ciudad Autónoma de Buenos Aires, Argentina). Each production batch of the ELISA was validated by the manufacturer using two EIAV-positive reference samples supplied by the USDA Animal and Plant Health Inspection Service (APHIS, Beltsville, MD, USA). For authorization of the commercial use of the ELISA in Spain, assay validation was performed using negative sera from internationally recognized disease-free regions and positive sera from infected animals, achieving 100% sensitivity and 99.9% specificity. Subsequently, in accordance with Law 8/2003 and Royal Decree 867/2020, the Spanish authorities approved and registered this serological assay for the detection of EIAV antibodies in equine serum and plasma. Seroprevalence was estimated as the proportion of seropositive animals among all tested individuals, with exact binomial 95% confidence intervals (95%CI) calculated according to [31]. A significance level of 0.05 was applied, and all statistical analyses were conducted using R software (version 4.5.1) [32].

## 3. Results and Discussion

None of the 1676 equines analyzed had antibodies against EIAV (0%; 95 %CI: 0.0–0.18). These results are consistent with the previously reported absent or low EIAV seroprevalence rates in Europe. Specifically, countries like Switzerland during 2007–2008 [20], Poland in 2018 [22], and Bulgaria [21] did not report EIAV circulation in the epidemiological studies conducted. Other serosurveys have detected low levels of virus circulation (<1%) in European equid populations, as observed in France during 2012–2014 [33,34], Serbia between 1994 and 2014 [35,36], Italy in 2007–2012 [37,38], and Hungary in 2008–2009 [39]. Conversely, higher EIAV exposure has been detected in some European countries such as Greece between 2001 and 2008 (4.5%; [40]), Belgium in 2007–2009 (6.3%; [25]), and Bosnia and Herzegovina in 2010 (12.3%; [24]).

Regarding continents other than Europe, previous epidemiological surveys have detected absent or low EIAV circulation in most of Asia, with Mongolia showing the highest EIAV prevalence in the continent (17.0%) [2,41]. In contrast, countries from America exhibit higher EIAV prevalence rates, as reported in donkeys from Mexico (22.0%; [42]) and in horses from Colombia in 2014 (22.9%; [43]), Guatemala in 2012 (40.6%; [44]), Brazil (46.3%; [45]), and Argentina in 2018 (100%; [46]).

The absence of EIAV circulation in the two Spanish regions sampled in the present study (0%; 0/1676; 95 %CI: 0.0–0.21) aligns with a previous serosurvey performed in this country during 2011–2013 [19]. That study analyzed 555 Spanish Purebred horses from provinces located in Central Spain (Madrid, Segovia, Avila, Cuenca, Guadalajara, and Toledo) and similarly found no evidence of EIAV circulation. Our results are also in agreement with the low number of EIA outbreaks (*n* = 2) reported in the country during the last decades, which were restricted to Western and Northwestern Spain during 2017–2018 (Caceres and Avila provinces, respectively) [26].

Regarding EIAV circulation in the UK (0%; 0/209; 95 %CI: 0.0–1.43) and Ireland (0%; 0/222; 95 %CI: 0.0–1.34), our findings revealed no detectable anti-EIAV antibodies in the equid populations analyzed. To the best of the author’s knowledge, this is the first epidemiological survey assessing EIAV circulation in these countries [2]. Of note, totals of 6 and 25 EIA outbreaks have been reported in the UK from 2006 to 2012 and in Ireland from 2006 to 2007, respectively [26]. The absence of EIA outbreaks in these countries during most of our study period aligns with the negative EIAV serological results obtained.

We did not find interspecific differences in EIAV exposure among equid species. However, previous studies evaluating the same species in different European countries have reported variable EIAV circulation among horses, donkeys, and mules [2]. For example, a study conducted in Greece in 2003 identified a higher EIAV seroprevalence in mules (15.3%) compared with donkeys (0.2%) and horses (0.0%) [23]. This species-dependent exposure of equids to EIAV has also been observed in Italy, where two previous serosurveys performed between 2007 and 2012 detected higher EIAV prevalence in mules (5.8–3.5%) than in horses (0.1–0.2%) and donkeys (0.1% in both) [37,38]. Similarly, Cappelli et al. (2011) [47] reported higher EIAV exposure in donkeys (25.0%) compared to mules (14.6%) and horses (12.9%) in Italy during 2006–2009. These findings suggest a higher infection risk in mules and donkeys compared to horses, emphasizing the need for future species-specific EIAV surveillance based on the virus’s multi-host tropism [2,48]. Although the reasons behind these interspecific differences remain unclear, similar patterns have been observed for other pathogens. For instance, studies conducted in Western Europe have reported higher exposure to *Toxoplasma gondii* and *Leishmania infantum* in mules and donkeys compared to horses [49,50]. These findings suggest that equid species maintained under extensive management systems (i.e., donkeys and mules) may be more exposed to infectious agents than horses, which are often kept under more controlled, indoor conditions. Moreover, the influence of additional factors such as immunological competence or genetic predisposition cannot be ruled out, since they may play a pivotal role in modulating responses to pathogens, potentially contributing to the interspecific differences observed [5,51]. Understanding how these individual factors influence pathogen transmission and disease progression could help refine species-specific surveillance and control strategies.

In this study, the limited sample size of donkeys and mules may constrain the strength of robust conclusions regarding differences in EIAV exposure found among equid species. Similarly, the use of convenience sampling could reduce the representativeness of the epidemiological information obtained. Consequently, future studies including larger, randomly selected, and more geographically diverse populations of equid species would be highly valuable.

The present large-scale study provides robust and up-to-date epidemiological evidence supporting the effectiveness of surveillance strategies and control programs implemented by European countries in accordance with Regulation (EU) 2016/429 [52] and complementary national measures [2]. The lack of updated information about EIAV circulation in most European countries highlights the need to promote serosurveys like ours in regions not covered by this study. This information will allow the acquisition of large-scale data on the health status of equids in Europe, as well as the establishment of evidence-based policies for accurate EIA management, if needed.

## 4. Conclusions

In conclusion, our results indicate the absence of EIAV circulation in horses, donkeys, and mules/hinnies from different regions of Spain, the UK, and Ireland. These findings provide strong evidence supporting the effectiveness of official surveillance programs, diagnostic protocols, and control measures implemented in these European countries, including biosecurity regulations, animal movement controls, or veterinary supervision, in preventing EIAV circulation. Nevertheless, the lack of recent scientific information on EIAV circulation in most European countries highlights a significant knowledge gap across the continent. Therefore, conducting comparable seroepidemiological studies in other regions would be essential to accurately assess the health status of European equid populations with respect to this notifiable disease.

Future research should focus on evaluating the potential for undetected EIAV circulation in Europe by incorporating molecular diagnostics to detect subclinical infections, assessing the use of additional EIAV antigens to enhance the sensitivity of serological assays and optimizing diagnostic protocols through the combined use of a complementary testing approach. Furthermore, identifying risk factors associated with the potential introduction and spread of EIAV into currently disease-free countries will be essential to inform evidence-based preventive policies and to promote coordinated surveillance efforts across Europe.

## Figures and Tables

**Table 1:** Frequency distribution of individual epidemiological variables in analyzed equids.

Variable	Categories	Number Equids (Relative Frequency %)
Study region	Andalusia	808 (48.2%)
	Catalonia	437 (26.1%)
	United Kingdom	209 (12.5%)
	Ireland	222 (13.2%)
Sex	Female	711 (46.2%)
	Male	828 (53.8%)
Breed	Pure	746 (53.8%)
	Crossbreed	641 (46.2%)
Age	Foal (<5 years)	221 (14.5%)
	Adult (5–14 years)	919 (60.1%)
	Geriatric (>14 years)	388 (25.4%)
Activity	Sport	263 (19.9%)
	Leisure	441 (33.3%)
	Reproduction	401 (30.3%)
	Work	218 (16.5%)
Body condition	Overweight	5 (0.4%)
	Adequate	1312 (97.4%)
	Thin	30 (2.2%)

## Data Availability

The data that support the findings of this study are available from the corresponding author upon reasonable request.

## Abbreviations

The following abbreviations are used in this manuscript:

EIA	Equine Infectious Anemia
EIAV	Equine Infectious Anemia Virus

## References

[1] Cook R.F., Leroux C., Issel C.J. (2013). Equine Infectious Anemia and Equine Infectious Anemia Virus in 2013: A review. Vet. Microbiol..

[2] Thieulent C.J., Carossino M., Reis J.K.P.D., Vissani M.A., Barrandeguy M.E., Valle-Casuso J.C., Balasuriya U.B.R. (2025). Equine Infectious Anemia virus worldwide prevalence: A 24-year retrospective review of a global equine health concern with far-reaching implications. Vet. Microbiol..

[3] Resende C.F., Santos A.M., Cook R.F., Victor R.M., Câmara R.J.F., Gonçalves G.P., Lima J.G., Maciel e Silva A.G., Leite R.C., dos Reis J.K.P. (2022). Low transmission rates of Equine Infectious Anemia Virus (EIAV) in foals born to seropositive feral mares inhabiting the Amazon Delta Region despite climatic conditions supporting high insect vector populations. BMC Vet. Res..

[4] Zhang B., Jin S., Jin J., Li F., Montelaro R.C. (2005). A tumor necrosis factor receptor family protein serves as a cellular receptor for the macrophage-tropic equine lentivirus. Proc. Natl. Acad. Sci. USA.

[5] Cook S.J., Cook R.F., Montelaro R.C., Issel C.J. (2001). Differential responses of *Equus caballus* and *Equus asinus* to infection with two pathogenic strains of Equine Infectious Anemia Virus. Vet. Microbiol..

[6] Issel C.J., Cook R.F., Mealey R.H., Horohov D.W. (2014). Equine Infectious Anemia in 2014: Live with it or eradicate it?. Vet. Clin. N. Am. Equine Pract..

[7] Sellon D.C., Fuller F.J., McGuire T.C. (1994). The immunopathogenesis of Equine Infectious Anemia Virus. Virus Res..

[8] Harrold S.M., Cook S.J., Cook R.F., Rushlow K.E., Issel C.J., Montelaro R.C. (2000). Tissue sites of persistent infection and active replication of Equine Infectious Anemia Virus during acute disease and asymptomatic infection in experimentally infected equids. J. Virol..

[9] Roberts H. (2017). Equine Infectious Anaemia in Europe: An ongoing threat to the UK. Vet. Rec..

[10] Lin Y.-Z., Shen R.X., Zhu Z.Y., Deng X.L., Cao X.Z., Wang X.F., Ma J., Jiang C.G., Zhao L.P., Lv X.L. (2011). An attenuated EIAV vaccine strain induces significantly different immune responses from its pathogenic parental strain although with similar in vivo replication pattern. Antivir. Res..

[11] WOAH (2019). Equine Infectious Anaemia—World Organisation for Animal Health. WOAH Terrestrial Manual.

[12] Hu Z., Chang H., Ge M., Lin Y., Wang X., Guo W., Wang X. (2014). Development of antigen capture ELISA for the quantification of EIAV P26 protein. Appl. Microbiol. Biotechnol..

[13] Russi R.C., Garcia L., Cámara M.S., Soutullo A.R. (2023). Validation of an indirect in-house ELISA using synthetic peptides to detect antibodies anti-gp90 and gp45 of the Equine Infectious Anaemia Virus. Equine Vet. J..

[14] Nagarajan M.M., Simard C. (2001). Detection of horses infected naturally with Equine Infectious Anemia Virus by nested polymerase chain reaction. J. Virol. Methods.

[15] Li S., Guo K., Wang X., Lin Y., Wang J., Wang Y., Du C., Hu Z., Wang X. (2023). Development and evaluation of a real-time quantitative PCR for the detection of Equine Infectious Anemia Virus. Microbiol. Spectr..

[16] Álvarez I., Cipolini F., Wigdorovitz A., Trono K., Barrandeguy M.E. (2015). The efficacy of ELISA commercial kits for the screening of Equine Infectious Anemia Virus infection. Rev. Argent. Microbiol..

[17] Lignee M. (1843). Memoire et Observations Sur Ure Maladie de Sarg, Connue Sous Le Nom d’anhemie Hydrohemie. Cachexie aqueuse du cheval. Rec. Med. Vet..

[18] Bolfa P., Barbuceanu F., Leau S.E., Leroux C. (2016). Equine Infectious Anaemia in Europe: Time to re-examine the efficacy of monitoring and control protocols?. Equine Vet. J..

[19] Cruz F., Fores P., Ireland J., Moreno M.A., Newton R. (2015). Freedom from Equine Infectious Anaemia Virus infection in Spanish purebred horses. Vet. Rec. Open..

[20] Kaiser A., Meier H.P., Doherr M.G., Perler L., Zanoni R., Gerber V. (2009). Serological and clinical proof of freedom from Equine Infectious Anemia (EIA) in imported and domestic horses in Switzerland. Schweiz. Arch. Tierheilkd..

[21] Chenchev I., Rusenova N., Sandev N. (2011). Seroepidemiological studies of donkeys’ blood for detection of some virus infections on ungulates. Trakia J. Sci..

[22] Bażanów B., Pawęska J.T., Pogorzelska A., Florek M., Frącka A., Gębarowski T., Chwirot W., Stygar D. (2021). Serological Evidence of common equine viral infections in a semi-isolated, unvaccinated population of Hucul horses. Animals.

[23] Spyrou V., Papanastassopoulou M., Koumbati M., Nikolakaki S.V., Koptopoulos G. (2005). Molecular analysis of the proviral DNA of Equine Infectious Anemia Virus in mules in Greece. Virus Res..

[24] Velić R., Velić L., Dukić B., Beširović H., Alić A., Zuko A., Omeragić J., Gušić A. (2011). Enzootic Infectious Anemia in Bosnia and Herzegovina in 2010. Vet. Stn..

[25] Caij A.B., Tignon M. (2014). Epidemiology and Genetic characterization of Equine Infectious Anaemia Virus strains isolated in Belgium in 2010. Transbound. Emerg. Dis..

[26] (2025). World Animal Health Information System (WAHIS). https://wahis.woah.org/#/event-management.

[27] (2025). International Collating Centre (ICC). https://equinesurveillance.org/iccview/.

[28] MAPA Programa de Vigilancia Fiebre Del Nilo Occidental 2025. https://www.mapa.gob.es/dam/mapa/contenido/ganaderia/temas/sanidad-animal-e-higiene-ganadera/sanidad-animal/enfermedades/enfermedades-que-son-comunes-a-varias-especies/fiebre-oeste-nilo/programafiebredelnilooccidental2025rev.pdfmapa.gob.es/dam/mapa/contenido/ganaderia/temas/sanidad-animal-e-higiene-ganadera/sanidad-animal/enfermedades/enfermedades-que-son-comunes-a-varias-especies/fiebre-oeste-nilo/programafiebredelnilooccidental2025rev.pdf.

[29] Franco J.J., Gonzálvez M., Cano-Terriza D., Barbero-Moyano J., Jose-Cunilleras E., Alguacil E., García J., García-Bocanegra I. (2025). Equine Viral Arteritis: Seroprevalence patterns and risk factors in equids from Western Europe. Res. Vet. Sci..

[30] Ostuni A., Frontoso R., Crudele M.A., Barca L., Amati M., Boni R., De Vendel J., Raimondi P., Bavoso A. (2025). Comparative evaluation of a multistrain indirect ELISA targeting anti-p26 and gp45 antibodies for EIAV detection. Pathogens.

[31] Thrusfield M., Christley R. (2018). Veterinary Epidemiology.

[32] (2025). R: A Language and Environment for Statistical Computing. R Core Team.

[33] Hans A., Dalgaz F., Lecouturier F., Courcoul A., Gay P., Gaudaire D., Grandcollot-Chabot M. (2014). Surveillance of Equine Infectious Anaemia: Two outbreaks detected in the south of France in 2014. Bulletin Épidémiologique, Animal Health and Nutrition No 71/Focus on Regulated and Emerging Diseases (REDs).

[34] Gaudaire D., Amelot G., Lecouturier F., Tapprest J., Hans A. (2016). Overview of surveillance of Equine Infectious Anaemia (EIA) in France in 2012. J. Equine Vet. Sci..

[35] Vidić B., Savić S., Grgić Ž., Bugarski D., Lupulović D., Prica N., Marčić D. (2014). Serosurveillance of Equine Infectious Anaemia in a region of Vojvodina. Arch. Vet. Med..

[36] Lupulovic D., Savić S., Gaudaire D., Berthet N., Grgić Ž., Matović K., Deshiere A., Hans A. (2021). Identification and genetic characterization of Equine Infectious Anemia Virus in Western Balkans. BMC Vet. Res..

[37] Maresca C., Scoccia E., Faccenda L., Zema J., Costarelli S. (2012). Equine Infectious Anemia: Active surveillance in Central Italy 2007-2009. J. Equine Vet. Sci..

[38] Carvelli A., Nardini R., Carnio A., Ricci I., Rosone F., Sala M., Simeoni S., Maccarone D., Scicluna M.T. (2024). Equine Infectious Anaemia: The active surveillance of an entire equid population reduces the occurrence of the infection. Transbound. Emerg. Dis..

[39] Rusvai M., Bakonyi T., HoarsiyAk A., Balka G., Hans A., Nowotny N. RT-PCR detection and phylogenetic analysis of Hungarian Equine Infectious Anemia Virus strains. Proceedings of the ESW 8th International Congress of Veterinary Virology.

[40] Mangana-Vougiouka O., Boutsini S., Ntousi D., Patakakis M., Orfanou E., Zafiropoulou K., Dilaveris D., Panagiotatos D., Nomikou K. (2013). Epizootiological investigation of the most important infectious equine diseases in Greece. Rev. Sci. Tech..

[41] Pagamjav O., Kobayashi K., Murakami H., Tabata Y., Miura Y., Boldbaatar B., Sentsui H. (2011). Serological survey of Equine Viral Diseases in Mongolia. Microbiol. Immunol..

[42] Villa-Mancera A., Villegas-Bello L., Campos-García H., Ortega-Vargas S., Cruz-Aviña J., Patricio-Martínez F., Olivares-Pérez J., Utrera-Quintana F. (2024). Prevalence of Equine Infectious Anemia Virus in horses and donkeys determined by comparison of ELISA and AGID in Mexico. Arq. Bras. Med. Vet. Zootec..

[43] Patiño B.E., Baldrich N.E., Malambo M.A., Parra W.D., Ortiz L.M., Patiño Herrera A. (2017). Report of gastrointestinal parasitosis and equine positive to equine infectious anemia in the animal health brigade in the year 2014 in the municipality of Florence-Caquetá. Rev. Electrón. Vet..

[44] Lepe-López M., García-Anleu R., Fountain-Jones N.M., Ponce G., Gonzalez M., Escobar L.E., Lepe-López M., García-Anleu R., Fountain-Jones N.M., Ponce G. (2018). Domestic horses within the Maya Biosphere reserve: A possible threat to the Central American Tapir (*Tapirus Bairdii*). Caldasia.

[45] Freitas N.F.Q.R., Oliveira C.M.C., Leite R.C., Reis J.K.P., Oliveira F.G., dos Bomjardim H.A., Salvarani F.M., Barbosa J.D. (2015). Equine Infectious Anemia on Marajo Island at the Mouth of the Amazon River. Pesqui. Veterinária Bras..

[46] Hébert L., Polledo G., Lecouturier F., Giorgi M., Beck C., Lowenski S., Laroucau K., Büscher P., Hans A., Becù T. (2021). Serological evidence of Equine Infectious Anaemia, West Nile Fever, Surra and equine piroplasmosis in a herd of horses in Northern Argentina. Vet. Parasitol. Reg. Stud. Rep..

[47] Cappelli K., Capomaccio S., Cook F.R., Felicetti M., Marenzoni M.L., Coppola G., Verini-Supplizi A., Coletti M., Passamonti F. (2011). Molecular detection, epidemiology, and genetic characterization of novel European field isolates of Equine Infectious Anemia Virus. J. Clin. Microbiol..

[48] Ataseven V.S., Arslan H.H. (2005). Equine Infectious Anemia in mules, donkeys, and horses: Epidemiologic studies in the different geographic regions of Turkey. J. Equine Vet. Sci..

[49] Cano-Terriza D., Franco J.J., Jose-Cunilleras E., Buono F., Almería S., Veneziano V., Alguacil E., García J., Villena I., Dubey J.P. (2023). Seroepidemiological Study of *Toxoplasma gondii* in equids in different European countries. Zoonoses Public Health.

[50] Barbero-Moyano J., Cano-Terriza D., Gonzálvez M., Moreno I., Jose-Cunilleras E., Buono F., Veneziano V., Alguacil E., García J., Veronesi F. (2025). Serosurvey of *Leishmania infantum* in equids in different European countries. Prev. Vet. Med..

[51] Jónsson H., Schubert M., Seguin-Orlando A., Ginolhac A., Petersen L., Fumagalli M., Albrechtsen A., Petersen B., Korneliussen T.S., Vilstrup J.T. (2014). Speciation with gene flow in equids despite extensive chromosomal plasticity. Proc. Natl. Acad. Sci. USA.

[52] (2016). Regulation (EU) 2016/429 of the European Parliament and of the Council of 9 March 2016 on Transmissible Animal Diseases and Amending and Repealing Certain Acts in the Area of Animal Health (‘Animal Health Law’).

